# Case report: Heart transplant for persistent right heart failure after complete surgical repair and percutaneous closure of post-myocardial infarction ventricular septal rupture

**DOI:** 10.3389/fcvm.2023.1237772

**Published:** 2023-09-18

**Authors:** Pankaj Garg, Amy Lykins, Mohammad Alomari, Peter Pollak, Parag Patel, Basar Sareyyupoglu

**Affiliations:** ^1^ Department of Cardiothoracic Surgery, Mayo Clinic, Jacksonville, Florida; ^2^ Department of Cardiovascular Diseases, Mayo Clinic, Jacksonville, Florida; ^3^ Department of Transplant, Division of Advanced Heart Failure and Cardiac Transplant, Mayo Clinic, Jacksonville, Florida

**Keywords:** heart transplant, ventricular septal rupture, structural heart disease, heart failure, right heart failure, acute myocardial infarction, mechanical circulatory support, cardiogenic shock

## Abstract

The incidence of post-acute myocardial infarction ventricular septal rupture (post-AMI VSR) has decreased; however, mortality after surgical repair of post-AMI VSR remains high. Patients who are not surgical candidates can be managed by heart transplant with a good outcome. A heart transplant in a patient after successful repair of VSR has never been reported. We report a patient who had persistent right heart failure after the successful repair of VSR and underwent a heart transplant with a good outcome.

## Introduction

Acute myocardial infarction (AMI) can result in mechanical complications e.g., ventricular septal rupture (VSR), papillary muscle rupture with severe mitral regurgitation and left ventricular free wall rupture. All these complications carry high mortality especially in patients with older age, female gender with hypertension and diabetes, anterior wall AMI, atypical symptoms of heart failure, and single vessel disease, particularly involving the left anterior descending coronary artery. Post-AMI VSR is a rare complication that develops within a few days after transmural AMI involving the septum. With improvement in the management of AMI, the incidence of post-AMI VSR has decreased to 0.17%–0.31% (1–6), but the mortality remains high. Apart from the development of a VSR, AMI also results in concomitant ventricular dysfunction in the territory of the involved coronary artery and can generate a macro-reentry circuit resulting in ventricular tachyarrhythmia and induce cardiogenic shock (CS) and low cardiac output due to large left to right shunt.

Surgical repair is the standard of care for these patients despite a 40%–50% mortality risk (5, 7), as medical management has a 30-day mortality approaching 100% for patients with large VSR with significant left to right shunt (1). Successful surgical repair of VSR results in obliteration of shunt and gradual improvement in ventricular function. However, patients with persistent right heart failure (RHF) after VSR repair are at increased mortality risk. Therefore, patients with post-AMI VSR need a multidisciplinary heart team approach. We report the management of a patient with posterior post-AMI VSR who had persistent RHF after complete surgical repair followed by percutaneous device closure of VSR and and successfully managed by an orthotopic heart transplant (OHT).

## Case report

**Figure 1:**
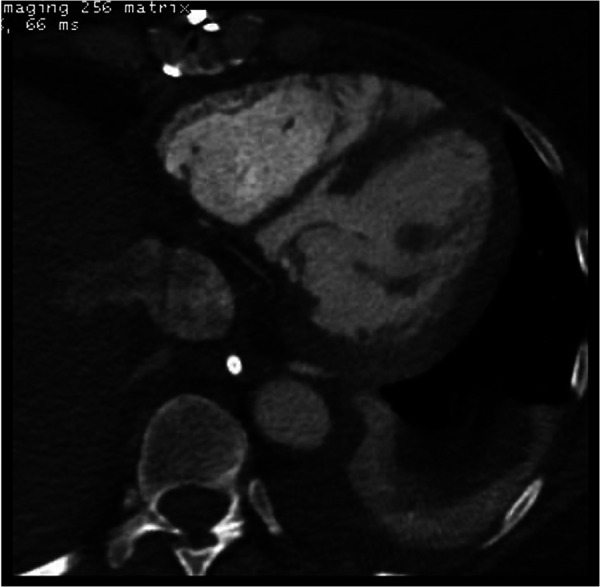
Contrast enhanced computed tomography of the chest performed 5 days after surgical repair of ventricular septal rupture (VSR) demonstrated the enlargement of small residual VSR.

**Figure 2:**
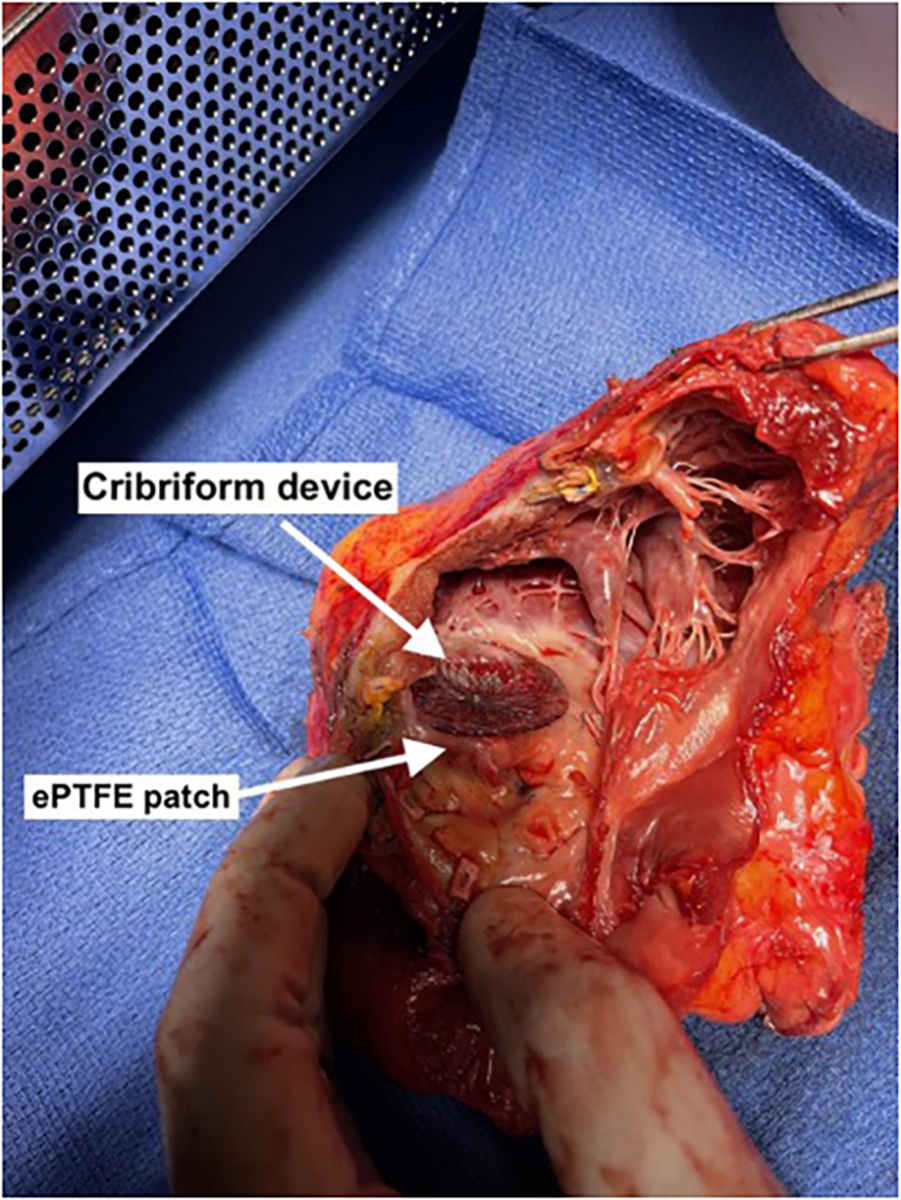
Cut open cardiectomy specimen ventricular septal device in place alongside expanded polytetrafluorethylene patch with complete closure of residual ventricular septal rupture.

**Figure 3:**
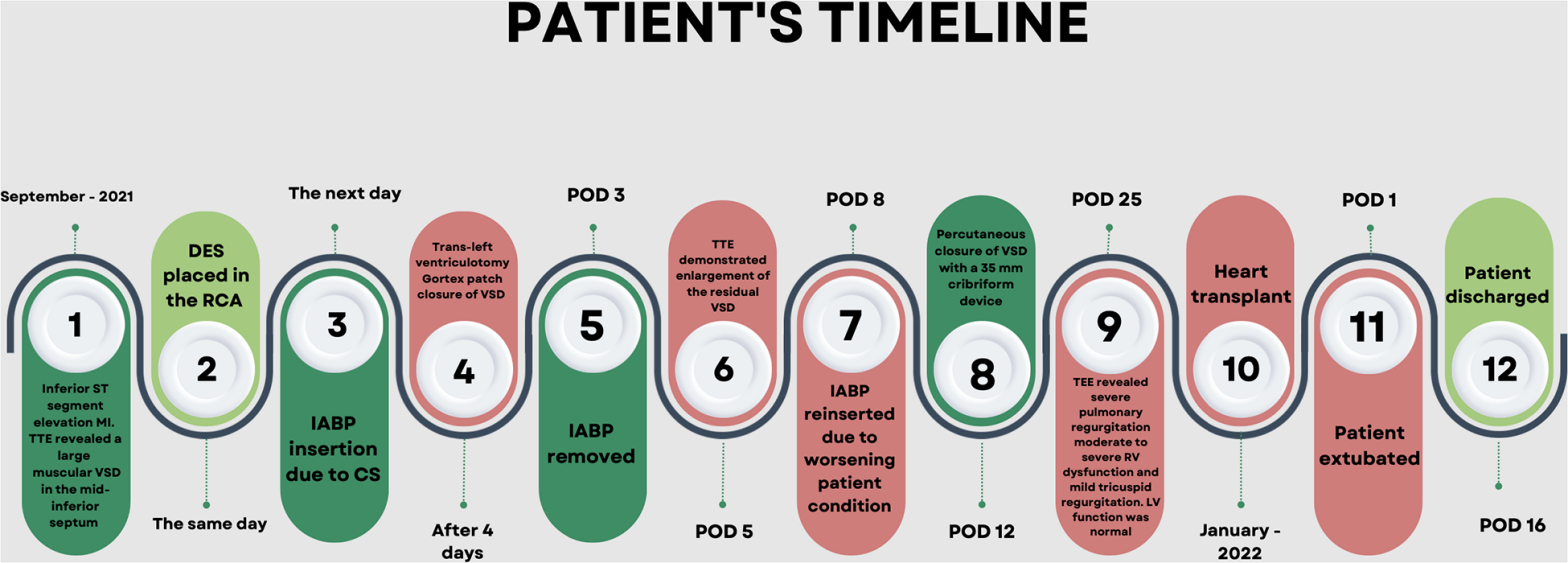
The timeline depicts the progression of patient's condition at various time points.

A 59-year-old African American female with a past medical history of hypertension and hyperlipidemia presented with inferior ST-segment elevation AMI. Her heart rate and rhythm were 100-110/min sinus, and her blood pressure was 90/60 mmHg. ECG revealed ST elevations in leads II, III, and AVF. Her Alanine Aminotransferase (ALT) and Aspartate Aminotransferase (AST) were 53 U/L and 77 U/L, respectively. Transthoracic echocardiography (TTE) revealed a large 16 mm muscular VSR in the mid-inferior septum with a significant left to right shunt and inferior wall hypokinesia (Supplementary Video S1). The left ventricular (LV) ejection fraction was 68%. Coronary angiography with right heart catheterization revealed a discrete 99% obstructive lesion in mid right coronary artery (RCA). Left main coronary artery was normal while there was a discrete 30% non-obstructive lesions in proximal left anterior descending and left circumflex coronary arteries. In right heart catheterization, cardiac index was 1.2L/min/m^2^; pulmonary artery wedge pressure (PCWP) was 21 mmHg, and pulmonary to systemic blood flow ratio (Qp:Qs) was 4:1. A drug-eluting stent (DES) was placed for stenosis in mid-RCA. Overnight, the patient developed hypotension, her ALT/AST increased to 445/420U/L, and lactate increased to 4.2 mmol/L. Intra-aortic balloon pump (IABP) was inserted the next day due to worsening CS. The patient was stabilized with IABP and inotropes but remained in CS. Four days later, the patient underwent surgical repair of VSR under moderate hypothermic cardioplegic arrest through median sternotomy. Intraoperatively, left ventriculotomy was performed parallel and 1–1.5 cm away from posterior descending coronary artery. The VSR was 2–2.5 cm in size, and the margins were necrotic. VSR was repaired with large polytetrafluroethylene (PTFE) patch using multiple interrupted 4-0 prolene sutures. Integrity of patch was confirmed with filling the right ventricle. There was minimal leak across the patch. Ventriculotomy was repaired over two strips of Teflon felts using interrupted 2-0 prolene sutures. After VSR repair, IABP was initiated, and the patient was weaned off cardiopulmonary bypass without difficulty. Postoperative transesophageal echocardiography showed trivial to mild residual VSR, moderate to severe RV dysfunction, and mild LV dysfunction. Post-VSR repair, the patient improved, and IABP was removed on postoperative day (POD) 3. TTE and CT (Figure 1) on POD 5 demonstrated enlargement of the residual VSR; however, the patient remained hemodynamically stable on minimal inotropes. On POD 8, the patient started to have decreased urine output. Therefore, IABP was reinserted, and dobutamine, milrinone, and oxygen were continued. On POD 12, the patient underwent percutaneous device closure of VSR with a 35 mm cribriform device in the catheterization lab. Right heart catheterization at the time of device closure revealed that right atrial pressure was 15 mmHg, mean pulmonary artery pressure 25 mmHg and PCWP 15 mmHg. Subsequent TTE showed good LV function, no residual VSR, and moderate to severe RV dysfunction with mild tricuspid regurgitation (Supplementary Video S2). IABP was removed on POD 16. The patient remained NYHA III and did not tolerate weaning from dobutamine, milrinone, and oxygen. Repeat TEE on POD 25 revealed severe pulmonary regurgitation with moderate to severe RV dysfunction and mild tricuspid regurgitation. LV function was normal. Although the etiology of severe pulmonary regurgitation was not clear, we believe that it was due to iatrogenic injury to the pulmonary leaflets during device closure of residual VSR. Due to persistent right heart failure and inotrope dependence, patient was reviewed by heart failure team and was listed for heart transplant. Four months after the first surgery, a heart became available, and the patient underwent an uneventful reoperative sternotomy, explantation of the native heart (Figure 2), and OHT. The patient did well in the postoperative period and was extubated on POD 1, transferred from the ICU on POD 4, and discharged from the hospital on POD 16. Timeline of events is shown in Figure 3. At last follow-up 18 months after OHT, patient was doing well. She remained NYHA class I with good biventricular function and no episode of allograft rejection.

## Discussion

In patients undergoing surgical repair of post-AMI VSR, complex and posterior VSR, emergency surgery, early repair, CS with hemodynamic instability, RV dysfunction, and renal impairment are risk factors for a poor outcome (8–11). Almost 50%–70% of patients with post-AMI VSR are in CS at the time of presentation, and more than 50% of these patients die due to multi-organ failure (12, 13).

Therefore, patients, who remain in CS despite IABP, and minimal inotropes should receive early escalation of mechanical circulatory support (MCS) instead of escalating the inotropes, which has detrimental effects on myocardial oxygen demand (14–17). Patients who fail to stabilize with IABP, early escalation of MCS to veno-arterial extra-corporeal membrane oxygenation should be considered (VA-ECMO). Care should be taken while considering VA-ECMO as it may significantly increase the afterload and left to right shunting across VSR. Stabilization of patient with MCS allows time for VSR margins to mature and makes the repair easier (7). Our patient had worsening CS on conservative management but stabilized after IABP insertion. Therefore, we did not consider escalating the MCS.

After managing CS, a decision should be made to manage the VSR. If possible, surgery should be delayed for one to two weeks as it allows time to mature VSR margin (7). However, decision should be based on clinical condition of the patient and operating surgeon's comfort as the MCS devices can improve the systemic perfusion but, no device is able to prevent left to right shunting. Patients who are surgery candidates based on the clinical condition and judgment of the operating surgeon based on the feasibility of VSR repair; should undergo surgical repair of VSR (1–4, 18). Despite successful surgical repair, almost 12%–20% of patients have residual VSR or develop a new VSR due to the extension of necrotic tissue or cutting through the VSR sutures. Residual VSR, however, has not been shown to increase mortality risk and can be safely closed with the device in the cath lab (19). Patients who are high-risk or not a surgery candidates should be evaluated for percutaneous device closure of VSR prior to considering for OHT. We performed the surgical repair of VSR as our patient was young, hemodynamically stable on IABP, had no co-morbidities, and VSR seemed amenable to closure on echocardiography. The patient had a good early result with a small residual VSR, and we removed the IABP on POD4. However, VSR increased in size over the next week with a recurrence of CS symptoms. The patient underwent successful device closure of residual VSR without any further leak.

In patients operated on for post-AMI VSR, right ventricle (RV) dysfunction and the posterior location of VSR significantly negatively impact both early and late survival (10, 12, 14, 15). RV infarct with sudden onset large left to right shunt across VSR results in RV stretching and dysfunction due to increased wall stress. Usually, RV dysfunction is reversible and may dictate the need for MCS in the perioperative period. Our patient had moderate to severe RV dysfunction in the immediate postoperative period and required continuation of IABP for MCS. The patient had some improvement in her RV function and was successfully weaned from IABP four days after surgery. However, due to the enlargement of residual VSR and severe pulmonary regurgitation, our patient continued to have severe RV dysfunction with symptoms of right heart failure. After evaluation by the heart failure team, it was decided to list the patient for a heart transplant.

The role of OHT as a primary or bailout strategy for patients with post-AMI VSR has been anecdotally described in case reports (20). All these patients were either supported with percutaneous MCS and listed for transplant or had implantable MCS and underwent a heart transplant. Unfortunately, none of these patients underwent successful VSR repair. Ours is the first case report of a heart transplant after the successful repair of VSR. Our patient underwent surgical repair of VSR followed by successful device closure of residual VSR but later underwent a heart transplant due to persistent right heart failure. Despite the successful outcome of OHT for majority of reported cases of post-AMI VSR, it should be considered as a last resort both because of the limited availability of donor hearts as well as patient has to on immunosuppressants for rest of their life. Further, heart failure should be adequately controlled with appropriate MCS device to improve the patient and allograft outcome after the OHT.

## Conclusions

Post-AMI VSR is rare but, devastating complication. Early recognition and management of mechanical complications of AMI is important to prevent poor outcome. Patients with large VSR with significant left to right shunt should be managed with appropriate mechanical circulatory support device to support the failing ventricle instead of escalating the inotropic support. If possible, repair should be delayed for one to two weeks. Patients with post-AMI VSR may continue to have heart failure after successful repair of VSR. Early recognition and appropriate management of right heart failure with mechanical circulatory support may prevent progressive right heart failure. Heart transplant should be considered as last resort and patient should be adequately managed for heart failure prior to listing for heart transplant. Patient with VSR who are adequately managed for heart failure can successfully undergo a heart transplant with a good outcome.

## Data Availability

The raw data supporting the conclusions of this article will be made available by the authors, without undue reservation.

## Supplementary material

Supplementary Video 1 Transthoracic echocardiography at the time of initial presentation revealed large basal ventricular septal rupture with left to right shunt.

Supplementary Video 2 Transthoracic echocardiography after cribriform device closure of the residual ventricular septal rupture (VSR) demonstrated normal left ventricular function, no residual VSR, and moderate to severe right ventricular dysfunction along with mild tricuspid regurgitation.
